# COVID-19 with Extreme Thrombocytosis: A Case Report and Its Possible Mechanisms

**DOI:** 10.22088/cjim.13.0.289

**Published:** 2022

**Authors:** Cathleen Kenya, Nur Chandra Bunawan, Hardijatmo Muljo Nugroho, Annisa Dian Harlivasari, Edgar David Sigarlaki, Ikhwan Rinaldi

**Affiliations:** 1 Regional Public Hospital of Kramat Jati, Jakarta, Indonesia; 2 COVID-19 Task Force in Regional Public Hospital of Kramat Jati, Jakarta, Indonesia; 3 Division of Hematology and Medical Oncology, Department of Internal Medicine, Faculty of Medicine Universitas Indonesia – dr. Cipto Mangunkusumo Hospital, Jakarta, Indonesia

**Keywords:** COVID-19, SARS-CoV-2, hematologic manifestations, platelet count, thrombocytosis

## Abstract

**Background:**

Coronavirus Disease 2019 (COVID-19) caused by Severe Acute Respiratory Syndrome Coronavirus-2 (SARS-CoV-2) has spread globally becoming a pandemic. The clinical manifestations of COVID-19 vary from asymptomatic to symptomatic disease. Hematologic manifestation which is commonly found in COVID-19 patients is thrombocytopenia whereas thrombocytosis is rarely reported.

**Case Presentation:**

We report a case of a 55-year-old woman with one week history of fever which spike along the day, dry cough, anosmia, nausea, epigastric pain and loss of appetite. She lived in local transmission area. The patient was diagnosed as mild suspected COVID-19 and confirmed with nasopharyngeal and oropharyngeal swab test (positive result). On admission, the number of platelet count was within normal limit but progressively increased exceeding 1000 x10^9^/L accompanied by worsening of the clinical condition. Interestingly, to our knowledge, no such case has ever been reported. In this study, we will discuss the possible mechanisms of its changes.

**Conclusions:**

COVID-19 can present with extreme thrombocytosis. Thus, monitoring the platelet count during hospitalization can be helpful for anticipating worsening conditions and progression of COVID-19.

A new coronavirus known as SARS-CoV-2 that emerged from Wuhan, China, has been reported to cause COVID-19. The numbers of cases are progressively increasing and it is rapidly spreading worldwide becoming a pandemic. In Indonesia, the first case was identified in March 2020. Currently, the cases had approached over several million infected people (1, 2). SARS-CoV-2 is a single-stranded RNA virus that can be transmitted among humans by direct contact or indirect contact, through contacting with any contaminated surfaces, or droplets of infected people (3).The mechanism that plays role in infecting human body is binding of SARS-CoV-2 to the angiotensin-converting enzyme 2 (ACE2) receptor. ACE2 receptor is widely expressed in alveolar epithelial cells of the lung, especially alveolar type II cells. In addition, ACE2 receptor is also found in the heart, endothelium of blood vessels, kidneys, and gastrointestinal tract so that multi-organ manifestations can occur in COVID-19 infection (4). According to Zhang et al.,ACE2 receptor is also expressed by the platelet where the virus could directly enhance platelet activation (5).The variety of clinical manifestations of COVID-19 ranges from asymptomatic to symptomatic infection, and is commonly manifested as a respiratory tract infection (4, 6).

According to the mechanism involved, it should be regarded as a systemic disease involving multiple systems including cardiovascular, respiratory, gastrointestinal, neurological, hematopoietic, and immune system (6, 7). Abnormal laboratory findings are commonly found in COVID-19, particularly hematological changes which include lymphopenia, neutrophilia, eosinopenia, mild thrombocytopenia and, less frequently, thrombocytosis (3). Patients with elevated platelet count had longer average hospitalization periods and worse outcomes. According to the previous studies, in severe COVID-19 the platelet significantly increased due to the cytokine storm. However, the underlying mechanisms remain unclear (6, 8). To our knowledge, extreme thrombocytosis has never been reported in COVID-19 patients. We reported a case of COVID-19 patient with extreme thrombocytosis during hospitalization and discussed its possible mechanisms.

## Case report

A 55-year-old woman refered to the emergency room presenting with fever one week prior to admission. The fever spike along the day and got better with antipyretic. The patient also experienced dry cough, anosmia, nausea, epigastric pain and loss of appetite. No shortness of breath, headache, ageusia, sore throat, and diarrhea were reported. Past medical history was positive for hypertension and well controlled with captopril 12.5mg OD. The patient denied any contact with COVID-19 positive patients but she lived in local transmission area.

**Figure 1:**
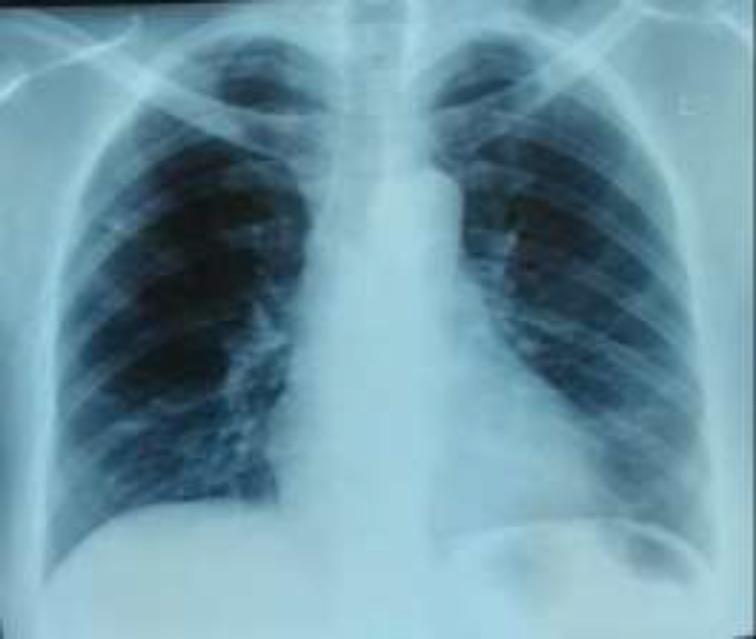
Chest X-ray on Admission

On admission, vital signs revealed blood pressure of 112/59 mmHg, temperature of 38.1^0^C, respiratory rate of 21 breaths per minute, heart rate of 71 beats per minute, and oxygen saturation of 99% on room air. Physical examination showed no rhonki/crackles/wheezing from lung and no murmur/gallop from heart. Abdominal examination revealed epigastric tenderness without organomegaly. No lymphadenopaty was noted. Laboratory findings on admission are shown in Table 1. Chest X-ray showed mild opacities in both lower-lobes (Figure 1) and normal ECG.

The patient was diagnosed as suspected mild COVID-19 at admission. The patient was given IV fluid, paracetamol 500mg tid (po), azitromisin 500mg OD (po), omeprazole 40mg bid (iv), ondancetron 4mg tid (iv), N-acetylsistein 200mg tid (po), sucralfat 15cc tid (po), zinc 20mg OD (po), and vit C 500mg OD (po). Nasopharyngeal and oropharyngeal swab test was taken and the result was positive for SARS-CoV-2 infection. Oseltamivir 75mg bid (po) was added to the regiment.

**Table 1:** Laboratory Findings on Admission

Parameter	**Result**
**Hemoglobin**	11.3 g/dL
**Hematocrit **	34%
**White Blood Cell**	8400 g/ul
**Platelet**	334 x 10^9^/L
**Segment**	64%
**Lymphocyte**	29%
**NLR**	2.21
**ALC**	2436 ul
**Blood Glucose**	130 mg/dl
**Sodium Serum**	139 mmol/L
**Potassium Serum**	3.9 mmol/L
**Chloride Serum**	106 mmol/L
**Blood Urea**	20 mg/dl
**Creatinine**	0.75 mg/dl
**AST**	22 mg/dl
**ALT**	15 mg/dl

NLR: Neutrophil-Lymphocyte Ratio; ALC: Absolute Lymphocyte Count; AST: Aspartate Transaminase; ALT: Alanin Aminotransferase

On Day-4 of hospitalization, patient’s conditions deteriorated.The fever reached40^0^C with worsening cough and shortness of breath. As a result, antibiotics were escalated to meropenem 1g tid (iv) and levofloxacin 750mg OD (iv). Oxygen was administered and uptitrated to 15 liters per minute of Non-Rebreathing Mask, anticoagulation withheparin 10.000 iu drip/24 hours was started, and the patient was transferred to Intensive Care Unit (ICU). 

On Day-6 of hospitalization, oxygenation of the patient couldn’t be achieved necessitating change to High Flow Nasal Cannula (HFNC) to maximum level of 60/60; antivirus was changed to Favipiravir. Colchicine 0.5mg bid (po), dexamethasone 5 mg tid (iv), and nebulized heparin 25.000 IU were also added to the treatment. In addition, heparin was increased to 15.000 iu/24 hours drip. On day-6 of hospitalization, blood samples were sent for culture. After the result was out, the organism was identified as Burkholderiacepacia. Therefore, Convalescent plasma transfusion was given twice, on day-7 and day-10 of hospitalization. Bilevel Positive Airway Pressure (BiPAP) was also administered on day-7, with PEEP 6, PS 8, FiO_2_ 80%. Patient’s condition improved on day-14 of hospitalization and BiPAP was switched to HFNC 40/30.

**Figure2:**
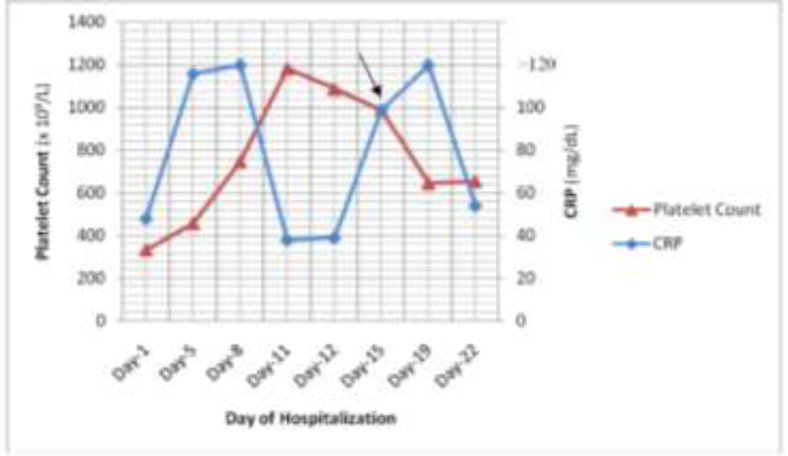
Platelet count profile, CRP and Hydroxyurea intervention during Hospitalization*

Simultaneously, along with the worsening of the patient’s condition, the platelet count started to rise on day-5 of hospitalization with peak of 1185 x 10^9^/L on day-11 of hospitalization.Hydroxyurea was administered 500mg bid (po) on day-15 of hospitalization and increased to tid (po) on the next day (Figure 2). Bone marrow puncture could not be performed due to our limitations.

*CRP: C-reactive Protein; The black arrow indicates the first intervention as the hydroxyurea firstly administered to the patient.

On day-18 of hospitalization, patient’s condition get worsen and NIV was readministered. On day-20 of hospitalization, urine was reported as sterile on culture. On day-22 of hospitalization, blood samples were reevaluated for culture. The organism was still identified as Burkholderia cepacia. Repeat Nasopharyngeal and oropharyngeal swab test were taken on day-17 and day-19 of hospitalization, which came back negative. However, intensive care unit management was still needed for the patient. Due to hospital limitations and regulations, COVID-19 patient with RT-PCR negative should be transferred to other facilities. Thus, the Patient was transferred to tertiary hospital in Jakarta, on day-24 of hospitalization. At the tertiary hospital, the patient was hospitalized for two days and intubated. Unfortunately, the patient had passed away due to worsening of the condition.

## Discussion

We reported a case of extreme thrombocytosis in COVID-19. Our patient didn’t have any history of increased platelet before, she had normal platelet count on admission which has progressively increased during hospital stays due to the worsening of her clinical condition. However, hematologic manifestation usually manifested in COVID-19 patients is thrombocytopenia instead of thrombocytosis. There were several possible mechanisms involved in thrombocytopenia, such as decreased platelet production, increased platelet destruction, and removal of circulating platelets. SARS-CoV-2 infection can directly infect the hematopoietic progenitor cells, megakaryocytes, platelet in bone marrow which leads to decrease number of platelet production. It can also induce autoantibodies or immune complexes that target platelets resulting in increased platelet destruction. Endothelial injury stimulates platelet activation and lung tissue injury entraps megakaryocytes preventing the release of platelets which may induce increased platelet removal from circulation (9). Since there have been no reports yet related to extreme thrombocytosis, we sought to review the available literature related to the possible mechanisms of extreme thrombocytosis in COVID-19.

SARS-CoV-2, which causes COVID-19, belongs to beta group of *Coronaviridae *family and it is the third notorious zoonotic disease of coronaviruses after Severe Acute Respiratory Syndrome (SARS) and Middle East Respiratory Syndrome (MERS) (10). The mechanisms involved in SARS-CoV-1 and SARS-CoV-2 for entering the host was through angiotensin converting enzyme 2 (ACE2) receptors (10, 11). There are many similarities in physical and chemical characteristics, but the affinity for human ACE2 was 10 times greater in SARS-CoV-2 compared to SARS-CoV-1 (10-12). There are 5 steps of the life cycle of the virus that invade the host: attachment, penetration, biosynthesis, maturation and release. Four structural proteins of coronaviruses are; Spike (S), membrane (M), envelop (E) and nucleocapsid (N) (13). After attaching to the ACE2 receptors, the spike proteins of SARS-CoV-2 are broken down through acid-dependent proteolysis by cathepsin, Transmembrane protease-serine 2 (TMPRSS2), or furin protease, and consequently, the SARS-CoV-2 merges with the cell membrane, which provides a strong option to adapt to its hosts. According to the previous studies, the spike protein could make a connection to Cluster of Differentiation 147(CD147) to invade the host cells. CD147 is a protein that expressed to a varying extent in the hematopoietic cells, mesenchymal stem cells, leukocytes, epithelial and endothelial cells, and has a wide range of physiological and pathological activities (10). 

There are three phases of SARS-CoV-2 infection:the initial phase caused by active infection, the second pulmonary phase, and the third hyperinflammatory phase characterized by cytokine storm. In some patients, the host inflammatory response continues to amplify and results in systemic inflammation (11, 14).

Early studies have shown that SARS patients had a high amount of proinflammatory cytokines in serum (e.g. interleukin-1B (IL1B), interleukin-6 (IL6), interleukin-12 (IL12), interferon-γ (IFNγ), IFNγ-inducible protein 10 (IP10), and monocyte chemoattractant protein 1 (MCP1)) which are associated with pulmonary inflammation and extensive lung damage. COVID-19 patients also had extensive pulmonary inflammation associated with high amount of proinflammatory cytokine.Moreover, patients requiring ICU admission had higher concentrations of granulocyte-colony stimulating factor (GCSF), IP10, MCP1, MIP1A, and tumor necrosis factor-α (TNFα) than those who didn’t require ICU admission, suggesting that the cytokine storm was associated with disease severity (15).

A variety of hematological changes in viral infection has been reported previously. According to a study by Raymond SM Wong et all.,which observed 157 SARS-CoV-1 infected patients, hematological changes found are thrombocytopenia (55%) with lowest platelet count at one week after the onset of symptoms, reactive thrombocytosis (49%) with a peak during the third week, whereas only one patient was found with extreme thrombocytosis and no evidence of thromboembolism (16). The platelet count peaked to 31 on day-6 of illness. MERS-CoV infection also associated with hematologic manifestations and mild thrombocytopenia was commonly found during the first week, without any difference between patients with mild or severe disease (17).

There are many definitions of thrombocytosis in the literature.Platelet count of ≥ 450 × 10^9^/L is a generally accepted value, while a platelet count of *>*1,000 *× *10^9^/L is defined as extreme thrombocytosis. The most common causes of thrombocytosis in adults were infection (typically acute), tissue damage, chronic inflammatory disorders, and malignancy (18). Secondary thrombocytosis is typically caused by the over-production of proinflammatory cytokines, especially IL-6 (19).

According to Shiyu et al., platelet count in COVID-19 group was significantly higher than non-COVID group, perhaps due to the reactively increased thrombopoietin (TPO) following pulmonary inflammation (20). Comparing the severity of the COVID-19 patients, the platelet peak was higher in severe patients who had longer hospitalization (12, 16, 20). A previous case report ona two-year-old girl with mild COVID-19 showed reactive thrombocytosis of peak platelet count 936 x 10^9^/L on day-6 of illness. She had normal platelet count on admission and never had any history of increased platelet count. Due to the improvement of her clinical condition, the platelet count decreased to 558 x 10^9^/L in the second week of COVID-19 illness (19).Reflecting to our case report, it was found that platelet count of the patient on the admission was within normal limit (334 x 10^9^/L) but progressively increased due to worsening of the condition related to cytokine storm. The platelet count peaked on day-11 of hospitalization (1185 x 10^9^/L).

Platelets are important immune cells in the human body, which play an important role in hemostasis, coagulation, vascular integrity maintenance, angiogenesis, mediators of inflammation and sensors of infectious agents through the interaction of cell surface receptors and pathogens (pathogen pattern recognition receptors) or immune system derivatives (immunoglobulin Fc receptors and complement receptors) (17,18). Platelets are produced by mature megakaryocytes in the bone marrow. Early studies have shown that elevated platelet counts could be an indicator of cytokine storm.Thrombopoietin (TPO), Interleukin-3 (IL-3), Interleukin-6 (IL-6), Interleukin-9 (IL-9), Interleukin-11 (IL-11) and stem cell factor (SCF) can promote the production of megakaryocytes. IL-3 and TPO is involved in the differentiation of megakaryocytes, while IL-6 is involved inpromoting the generation of megakaryocytes through stimulation of TPO leading to increased platelet synthesis. Another possible mechanism involved starts from endothelial injury which stimulates the release of von willebrand factor (vWF). vWF may interact with megakaryocytes via binding to platelet membrane glycoprotein Ib (GPIbvWF) leading to an increase in platelet production. TPO also plays a role in stimulating lung megakaryocytes to produce platelets (9, 18). In accordance with Huang et al studies, the number of platelet count significantly increased during treatment due to release of a high amount of cytokines by the immune system. COVID-19 causes cytokine storms in body fluids, aggravating the patient's inflammatory response and stimulating the release of platelets, which often indicates a poor prognosis. However, there are many factors that affect platelet changes in the clinic. Circulating levels of high amount of cytokines, particularly IL-6 are elevated in patients with reactive thrombocytosis.There are speculation that changes in platelet count could indicate disease progression and prognosis of COVID-19 patients. Elevated of platelet counts pertained to poor prognosis and worsening condition (12, 18).

Many other markers of the acute phase reaction, including C-reactive protein (CRP) are also significantly elevated in patients with reactive thrombocytosis. According to Tefferi et al study., 76% of patients with reactive thrombocytosis had an elevated CRP (>1.0 mg/dL). Thus, measurement of CRP can be easily applied for measurement of cytokines in thrombocytopoiesis and thus should be a part of any evaluation where reactive thrombocytosis is suspected (18).

Hydrea^®^ (Hydroxyurea) was administered on day-15 of hospitalization due to constantly high number of platelet counts after several days of repeated blood count. Hydroxyurea is an antimetabolite which primarily acts in S phase and is effective in reducing platelet counts. Dosage of hydroxyurea was titrated to maintain a platelet count <600 x 10^9^/L (18). It gave a positive feedback as the number of platelet count of the patient progressively decreased though the CRP was rising again (Figure 2).

In conclusions although the common hematologic manifestations in COVID-19 seem to be thrombocytopenia, it can also manifest as extreme thrombocytosis. Extreme thrombocytosis is attributed to the high number of cytokine released and severity of the disease. Monitoring of blood count periodically is recommended as an indicator for strict observation anticipating worsening conditions and progression of COVID-19 patients. However, further studies are necessary to investigate the exact mechanisms of this clinical conundrum.
